# Essential Oil Variation from Twenty Two Genotypes of *Citrus* in Brazil—Chemometric Approach and Repellency Against *Diaphorina citri* Kuwayama

**DOI:** 10.3390/molecules21060814

**Published:** 2016-06-22

**Authors:** Moacir dos Santos Andrade, Leandro do Prado Ribeiro, Paulo Cesar Borgoni, Maria Fátima das Graças Fernandes da Silva, Moacir Rossi Forim, João Batista Fernandes, Paulo Cezar Vieira, José Djair Vendramin, Marcos Antônio Machado

**Affiliations:** 1 Departamento de Química, Universidade Federal de São Carlos, CP 676, São Carlos-SP 13565-905, Brazil; msandrade2003@gmail.com (M.S.A.); mrforim@gmail.com (M.R.F.); djb@ufscar.br (J.B.F.); dpcv@ufscar.br (P.C.V.); 2 Departamento de Entomologia e Acarologia, Universidade de São Paulo, Escola Superior de Agricultura Luiz de Queiroz (USP/ESALQ)-Av. Pádua Dias, 11, Agronomia, CEP 13418-900, Piracicaba-SP, Brazil; leandroribeiro@epagri.sc.gov.br (L.P.R.); pcbogorni@yahoo.com.br (P.C.B.); jdvendra@usp.br (J.D.V.); 3 Centro APTA Citros Sylvio Moreira, Instituto Agronômico, CP 04, Cordeirópolis-SP 13490-970, Brazil; marcos@centrodecitricultura.br

**Keywords:** *Citrus*, *C. reticulate*, *C. sinensis*, *Murraya paniculata*, *Diaphorina citri*, Huanglongbing, essential oil, olfactory, chemometrics, GC-MS

## Abstract

The chemical composition of volatile oils from 22 genotypes of *Citrus* and related genera was poorly differentiated, but chemometric techniques have clarified the relationships between the 22 genotypes, and allowed us to understand their resistance to *D. citri*. The most convincing similarities include the synthesis of (*Z*)-β-ocimene and (*E*)-caryophyllene for all 11 genotypes of group A. Genotypes of group B are not uniformly characterized by essential oil compounds. When stimulated with odor sources of 22 genotypes in a Y-tube olfactometer *D. citri* preferentially entered the arm containing the volatile oils of *Murraya paniculata*, confirming orange jasmine as its best host. *C. reticulata* × *C. sinensis* was the least preferred genotype, and is characterized by the presence of phytol, (*Z*)-β-ocimene, and β-elemene, which were not found in the most preferred genotype. We speculate that these three compounds may act as a repellent, making these oils less attractive to *D. citri*.

## 1. Introduction

Pesticides are the second most important item in the import account of the agricultural sector in Brazil, behind only fertilisers. The sale of pesticides in Brazil will be aided by increases in one of the most serious diseases of *Citrus*, Huanglongbing (HLB), previously called greening. HLB is a disease that has been correlated with the phloem-restricted bacteria *Candidatus* Liberibacter spp [1,2]. Both *Ca*. Liberibacter asiaticus and *Ca.* L. americanus are associated with HLB in Brazil. This disease is the main cause of reduced yields in *Citrus* orchards in Africa, Asia and Brazil and can lead to a total loss of production [3].

The bacteria associated with this disease are transmitted by the Asian *Citrus* psyllid, *Diaphorina citri* Kuwayama, which is common in Brazilian orchards [4]. The insect is also common and usually abundant on the exotic ornamental plant, orange jasmine (*M. paniculata*, *Sapindales*, *Rutaceae*), which is considered one of its best hosts. In addition to being the HLB vector, *D. citri* is a sap-sucking insect that can cause damage such as leaf curling and twisting of shoots. If the infestation is intense, the stems dry up and the year’s production may suffer substantial reduction. HLB was detected for the first time in Brazil in orchards in the state of São Paulo in July of 2004 [5]. However, HLB was probably already present for a long time in Brazil, explaining the wide spatial extension of the disease observed today, and requiring insecticide application to prevent plant damage.

The above data demonstrate the great need for a program for the minor use of pesticides. Thus, the development of effective alternatives to conventional insecticides for management of *D. citri* is of critical importance. Research has shown genetic variability in resistance to *D. citri* in *Citrus* species and other *Rutaceae* species used by psyllids as host plants [6,7,8,9,10,11]. Resistant *Citrus* genotypes may prevent the colonization or multiplication of *D. citri*, but some of them are not close to commercial quality. Therefore, it has become important to find relatively easy alternative control strategies that are as effective as synthetic pesticides but are safer for the farmers, consumers, and environment and are cost effective. One of the possible alternatives would be the use of pesticides or insect repellents of plant origin, as from *Citrus* genotypes resistant or less preferred hosts for *D. citri*.

Essential oils from plants generally are active as insect attractants or repellents. Although the mechanisms of action of insect repellents may differ greatly and are often not yet well understood, they have the advantage of combining a wide range of toxic potencies, thereby reducing the chance of pests developing resistance [12]. In addition, residues are hardly expected on the products or in the environment because essential oils are generally non-persistent under field conditions, as they are readily degraded by light and oxygen into less toxic products. Brazil is in a good position to develop and utilise plant-derived essential oils because this country has a rich biodiversity of *Citrus* species.

Borgoni and collaborators evaluated eight genotypes of *Citrus* and related genera (‘Pera’, ‘Natal’, and ‘Washington Navel’ oranges (*C. sinensis*), ‘Marsh Seedless’ grapefruit (*C. paradisi*), hardy orange ‘Rubidoux’ (*Poncirus trifoliata*), kumquat (*Fortunella margarita* Swingle), citrumelo ‘Swingle’ (*C. paradisi* × *P. trifoliata*), and citrange ‘Troyer’ (*P. trifoliata* × *C. sinensis*)) by determining whether they influence oviposition of *D. citri* in no-choice tests [13]. The experiments were performed in greenhouses with plants grafted onto ‘Rangpur’ lime (*C. limonia*) and placed individually in cheesecloth cages. ‘Rubidoux’ (*P. trifoliata*) was the least preferred genotype for oviposition; reduced number of eggs was also found on citrange ‘Troyer’, while ‘Marsh Seedless’ was the genotype with the most eggs. Considering all of the evaluated parameters, it was concluded that cultivars of sweet orange are the most susceptible genotypes to *D. citri*. Regarding oviposition, *P. trifoliata* ‘Rubidoux’ showed resistance of the antixenosis type.

*Diaphorina citri* female oviposits and develops exclusively on new shoots; thus, its life cycle is closely tied to the growing pattern of its host plants [14]. Some studies have shown that stimuli emitted by new shoots may play an important role in the detection, location, and evaluation of potential host plants by Asian *Citrus* psyllid. We have now examined the essential oil of new shoots from the eight genotypes of *Citrus* cited above in order to determine if compounds present in the oil explain the preference of *D. citri* to sweet orange cultivars and non-preference to *P. trifoliata* ‘Rubidoux’ for oviposition. However, the overall aim of this study was to identify potential volatile compounds of 22 genotypes of *Citrus*, which could be used to develop olfactory lures for enhancing *D. citri* detection and monitoring, as well as to identify effective repellents from plants.

## 2. Results and Discussion

**Figure 1:**
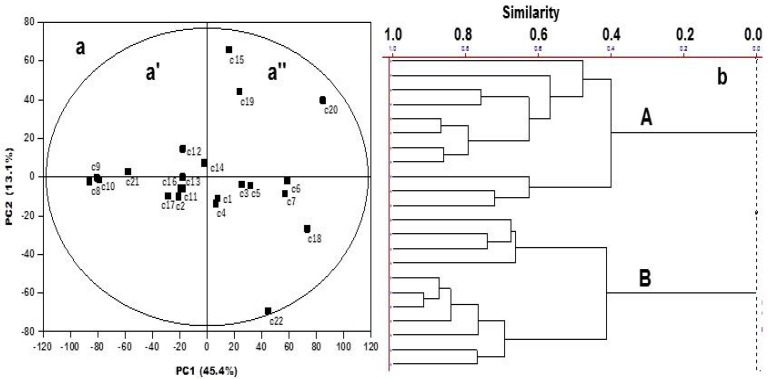
HCA and PCA analyses of Citrus genotypes. (**a**) The score scatterplot obtained according to the two major groups and (**b**) dendrogram based on GC-MS data of 22 volatile oils from 22 genotypes of *Citrus*.

Compositions of the essential oils are given in Table S1. Component concentrations were calculated from GC peak areas and they were arranged in order of GC (DB-5) elution. The chemical compositions of the volatile oils from the 22 genotypes of *Citrus* and related genera were poorly differentiated (Table S1). Such deductions were based primarily on the evaluation of chemical compounds as markers following the static presence and absence criterion. Within this context, the purpose of the present study was to examine the chemical composition of volatile oils in a dynamic approach, as chemometric techniques, in order to check and clarify the relationships between the 22 genotypes, and if possible to understand their resistance to *D. citri*. The dendrogram of the 22 genotypes using two PCs is presented in Figure 1b and the corresponding score scatter plot in Figure 1a. The dendrogram differentiates two major groups (A and B) within the dataset which correspond to the two highlighted clusters a′ and a″ in the scores plot (Figure 1a).

**Figure 2:**
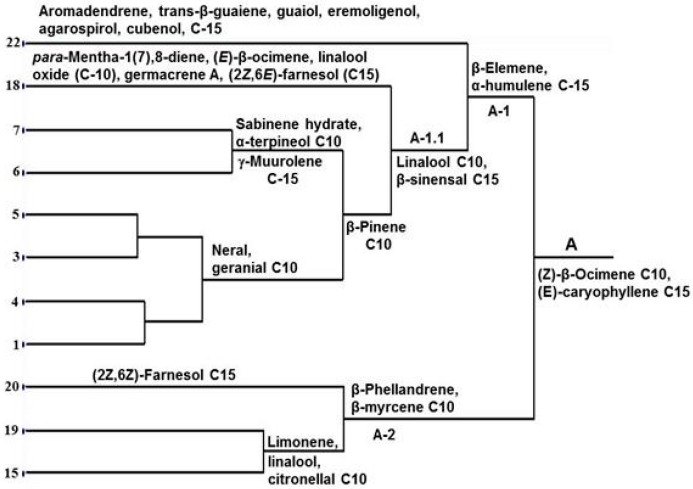
Chemical similarities between 11 genotypes of group A. Number at end of each dendrogram line refers to *Citrus* genotype (C-1–C-22: see Material and Methods—Plant material).

Figure 2 summarizes some chemical evidence supporting similarities between the 11 genotypes of group A. The most convincing similarities include the synthesis of (*Z*)-β-ocimene and (*E*)-caryophyllene for all 11 genotypes. Within this group are two subgroups, one having in common β-elemene and α-humulene (A-1), which also occur in members belonging to the second subgroup (A-2) (Table S1). However, the co-occurrence of β-phellandrene and β-myrcene in *P. trifoliata* (C-20), *C. sunki* × *P. trifoliata* (C-19) and *C. medica* (C-15) convincingly point to true similarities between these three genotypes (A-2).

The chemical composition of volatile oils of group A-1 suggests the early separation of *F. margarita* (C-22) with aromadendrene, *trans*-β-guaiene, guaiol, eremoligenol, agarospirol and cubenol absent in the remaining genotypes of this group. At this stage the dendrogram leads to a second subgroup A-1.1 sharing linalool and β-sinensal, but *P. trifoliata* × *C. sinensis* (C-18) taking an isolated position with *para*-mentha-1(7),8-diene, (*E*)-β-ocimene, linalool oxide, germacrene A and (2*Z*,6*E*)-farnesol, which so far were not detected in this group. The remaining 6 genotypes all containing β-pinene are subdivided into two subgroups, one having in common sabinene hydrate, α-terpineol and γ-muurolene (consists of *C. reticulata* (Pokan C-7 and Cravo C-6)), and the other neral and geranial. This last one is subdivided in two including *C. sinensis* (Hamlin C-5 and Valencia C-3), and *C. sinensis* (Washington Navel C-4 and Pera C-1), respectively. There are no other comparable distinctions that can be drawn between C-5, C-3, C-4 and C-1 as a unit; they are characterized by some types of mono- and sesquiterpenes found in other groups. In group A-2 (2*Z*,6*Z*)-farnesol is restricted to C-20, while limonene, linalool and citronellal occur in C-19 and C-15.

**Figure 3:**
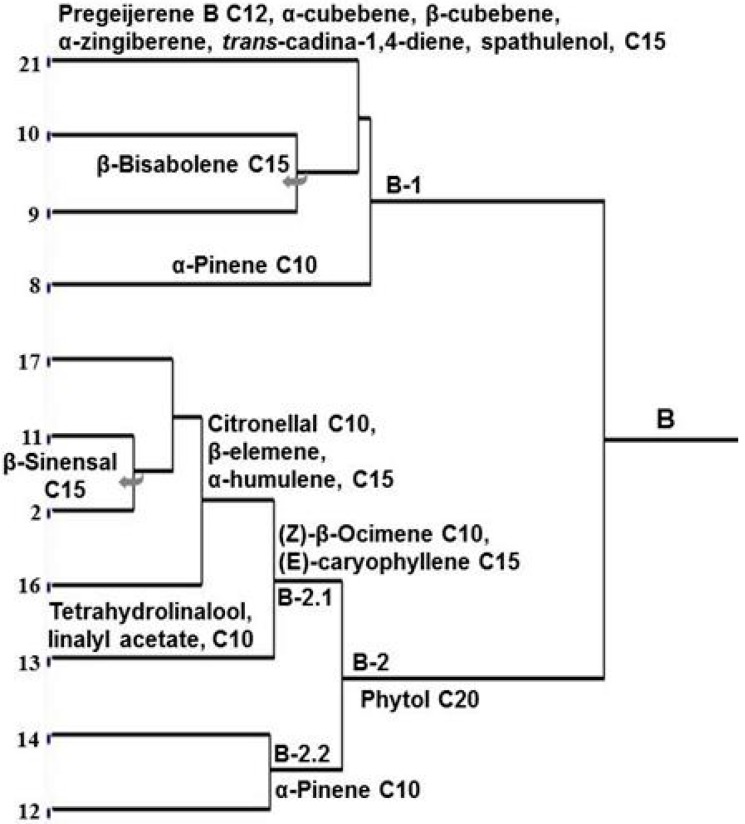
Chemical similarities between 11 genotypes of group B. Number at end of each dendrogram line refers to *Citrus* genotype (C-1–C-22: see Material and Methods – Plant material).

Genotypes of group B (Figure 3) are not uniformly characterized by essential oil compounds. They are subdivided into two subgroups B-1 and B-2, the first being heterogeneous. In subgroup B-1 the early separation of *C. deliciosa* (C-8) is justified by the presence of α-pinene which occurs only in members of B-1. Pregeijerene B, α-cubebene, β-cubebene, α-zingiberene, *trans*-cadina-1,4-diene and spathulenol are restricted to *M. paniculata* (C-21), and β-bisabolene to *C. latifolia* (C-10) and *C. limettioides* (C-9). Subgroup B-2 has in common phytol and then it is subdivided in B-2.1 and B-2.2, and the last is small with *C. grandis* (C-14) and *C. limon* (C-12) sharing α-pinene. In spite of great diversity in B-2.1, they share (*Z*)-β-ocimene and (*E*)-caryophyllene, and at least two subdivisions can be recognized: (a) *C. aurantium* (C-13) with tetrahydrolinalool and linalyl acetate, which only occur in this genotype, and (b) *C. paradisi* × *P. trifoliata* (C-17), *C. paradisi* (C-11), *C. sinensis* (Natal C-2) and *C. reticulata* × *C. sinensis* (C-16) sharing citronellal, β-elemene and α-humulene. In addition, C-11 and C-2 share together β-sinensal.

This interpretation of chemical data is consistent with morphology ones for *C. sinensis* and *C. reticulata*. Chemical characters link the four cultivars of *C. sinensis* (Hamlin C-5, Valencia C-3, Washington Navel C-4 and Pera C-1), and two of *C. reticulata* (Pokan C-7 and Cravo C-6) (Figure 2). *C. sinensis* cultivar Natal (C-2) is an exception; its essential oil compounds are similar to those from *C. paradisi* (C-11) and the hybrids *C. reticulata* × *C. sinensis* (C-16) and *C. paradisi* × *P. trifoliata* (C-17). However, *C. sinensis* cultivar Natal (C-2) also has in common many mono- and sesquiterpenes with the other cultivars (C-5, C-3, C-4 and C-1; Table S1). Furthermore, it is obvious that the hybrid *C. reticulata* × *C. sinensis* (C-16) is much more similar to *C. sinensis* cultivar Natal than to the other cultivars Hamlin C-5, Valencia C-3, Washington Navel C-4 and Pera C-1. The data also convincingly point to greater similarities between the hybrid *C. paradisi* × *P. trifoliata* (C-17) and *C. paradisi* (C-11) than with *P. trifoliata* (C-20). Instead, the hybrids *P. trifoliata* × *C. sinensis* (C-18) and *C. sunki* × *P. trifoliata* (C-19) shares a sufficient number of mono- and sequiterpenes to be included near to *P. trifoliata* (C-20) and *C. sinensis* (Hamlin C-5, Valencia C-3, Washington Navel C-4 and Pera C-1).

**Figure 4:**
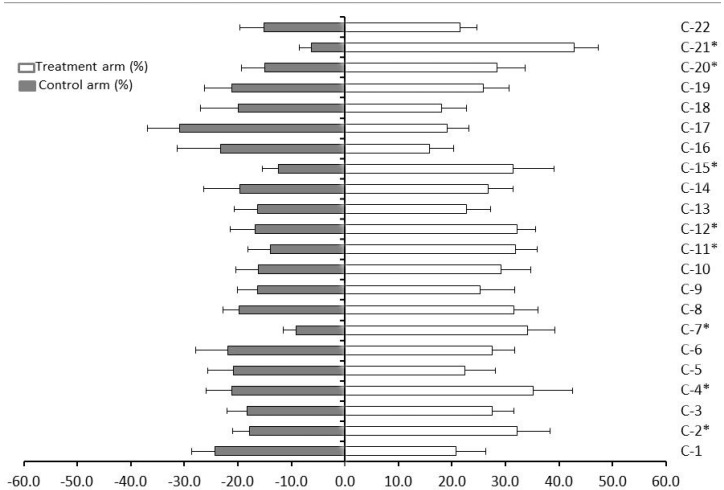
Response of adult psyllids to 22 volatile oils from 22 genotypes of *Citrus*. Asterisks denote statistically significant difference in response between the test and control odor, Chisquare, *p* ≤ 0.05.

The responses of *D. citri* males and females in a Y-tube olfactometer when they were stimulated with odor sources of 22 volatile oils from 22 genotypes of *Citrus* are shown in Figure 4. In the preliminary test with the blank air, no significant differences were obtained between the arms of the olfactometer, with about 50% of insects entering in both arms. These results showed that both arms were equivalent and did not introduce any bias during the choice tests with host plant volatile oils. More than 70% of *D. citri* entered either arm of the olfactometer, indicating that most individuals tested were in a responsive condition. *D. citri* preferentially entered and remained in the arm containing the odor from volatile oils of *M. paniculata* (C-21) over all the 21 volatile oils and unscented control, confirming orange jasmine as its best host (Figure 4). *C. reticulata* × *C. sinensis* (tangor) ‘Murcott’ (C-16) was the least preferred genotype (15.7%). Reduced choice was also found to occur in 09 volatile oils from, in order of their decreasing preference, *C. grandis* (C-14, 26.8%), *C. sunki* × *P. trifoliata* (C-19, 25.9%), *C. limettioides* (C-9, 25.3%), *C. aurantium* (C-13, 22.8%), *C. sinensis* cultivar Hamlin (C-5, 22.4%), *F. margarita* (C-22, 21.6%), *C. sinensis* cultivar Pera (C-1, 20.8%), *C. paradisi* × *P. trifoliata* (C-17, 19.1%), and *P. trifoliata* × *C. sinensis* (C-18, 18.1%). The least preferred genotypes have in common phytol, and except *C. grandis* (C14), they also share (*Z*)-β-ocimene (Table S1).

(*E*)-caryophyllene occurs in the last 08 genotypes of low preference (C-13, C-5, C-22, C-1, C-17, C-18 and C-16), and β-elemene and α-humulene were obtained from all except in C-13. The most interesting is that phytol, (*Z*)-β-ocimene and β-elemene were not found in the most preferred genotype *M. paniculata* (C-21). We speculate that these three compounds may act as a repellent, making these oils less attractive to *D. citri*.

*Citrus paradisi* × *P. trifoliata* (C-17), *C. reticulata* × *C. sinensis* (tangor) ‘Murcott’ (C-16), *C. sinensis* cultivar Pera (C-1), and *P. trifoliata* × *C. sinensis* (C-18) exhibit moderate repellency against *D. citri*, showing index of repellency (IR) values of 0.76, 0.81, 0.92 and 0.95, respectively (Table 1). The repellency index is classified as: values <1 repellency; 1 neutral; >1 attractant (see materials and methods) [15]. Thus, the least preferred genotype C-16 (15.7%; IR 0.81) is less repellent than C-17 (19.1%; IR 0.76).

The Y-tube olfactory data also support the results regarding oviposition obtained by Borgoni *et al.*, who found that the number of eggs oviposited on *P. trifoliata* × *C. sinensis* (C-18) was significantly less than on either *C. sinensis* Washington Navel (C-4) or *C. paradisi* (C-11) [13]. It is not clear why *D. citri* were not less attracted to *P. trifoliata* (C-20) in the Y-tube tests, because this genotype was the least preferred for oviposition in a previous study [13]. However, phytol (12.1%), (*Z*)-β-ocimene (0.9%) and β-elemene (4.1%) occur also in *P. trifoliata* (C-20). Only β-elemene was observed at a higher percentage in *P. trifoliata* × *C. sinensis* (C-18) and *C. reticulata* × *C. sinensis* (tangor) ‘Murcott’ (C-16), 13.4 and 16.6% respectively. ‘Murcott’ (C-16) was not included in the Borgoni *et al.* study [13]. One might expect oviposition to be cued largely by volatile chemicals but this is not so; non-volatile chemicals are equally well represented. Volatile compounds play an important role in attracting *D. citri* to their chosen host plant for both feeding and oviposition, so perhaps some *P. trifoliata* (C-20) volatile compounds attract *D. citri*, but non-volatile oviposition stimulants were lacking. For example, *Papilio xuthus* L. (Papilionidae) is a swallowtail butterfly whose larvae feed exclusively on plants in the family *Rutaceae* [16]. More than seventy related species in the genus *Papilio* are strongly associated with rutaceous plants and many of them are pests of *Citrus* crops. The females lay eggs with great precision on young leaves of their host plants. The oviposition stimulant of *P. xuthus* appeared to be a mixture of highly polar non-volatile compounds.

The least preference of *D. citri* for *C. reticulata* × *C. sinensis* (tangor) ‘Murcott’ (C-16), may well be owing to the presence of phytol, (*Z*)-β-ocimene and β-elemene. However, it is premature to draw any conclusion about the resistance to oviposition by *D. citri*, until ‘Murcott’ can be evaluated in oviposition tests. Greenhouse experiments were conducted by Lopes and Frare with the objective of determining conditions favorable for transmission from field affected trees to young potted plants [17], to evaluate the reaction of multiple *Citrus* species to the disease, and to determine the efficiency of pathogen propagation from individual buds. Ca. Liberibacter americanus’ was transmitted at higher percentages to sweet oranges (31.2% to 65.2%) and ‘Murcott’ (44.4%). However, if *D. citri* prefers ‘Murcott’ as a host in the field this has not been widely documented to date.

## 3. Materials and Methods

### 3.1. Plant Material

The 22 genotypes of *Citrus* were obtained from the active germoplasm bank of the Centro APTA Citros Sylvio Moreira (Cordeirópolis, SP-Brazil): *C. sinensis* (sweet orange) cv. ‘Pera’ (C-1), ‘Natal’ (C-2), ‘Valencia’ (C-3), ‘Washington Navel’ (‘Bahia’) (C-4) and ‘Hamlin’ (C-5); *C. reticulata* Blanco (tangerine or mandarin) cv. ‘Cravo’ (C-6) and ‘Ponkan’ (C-7); *C. deliciosa* Tenore (mandarin) cv. ‘Mexerica-do-rio’ (C-8); *C. limettioides* Tanaka (sweet lime) cv ‘Palestine’ (C-9); *C. latifolia* Tanaka (lime) cv. ‘Tahiti’ (C-10); *C. paradisi* Mcf. (grapefruit) cv. ‘Marsh Seedless’ (C-11); *C. limon* (L.) Burm. F. (Sicilian lemon) (C-12); *C. aurantium* L. (sour orange) (C-13); *C. grandis* Osbeck (sweet pummel) (C-14); *C. medica* L. (citron) (C-15); *C. reticulata* L. × *C. sinensis* L. (tangor) ‘Murcott’ (C-16); *C. paradisi* × *P. trifoliata* (citrumelo) cv. ‘Swingle’ (C-17); *P. trifoliata* × *C. sinensis* (citrange) cv. ‘Troyer’ (C-18); *C. sunki* hort. ex Tanaka × *P. trifoliata* L. Raf. (citrandarin) ‘English’ (C-19); *P. trifoliata* L. Raf. (poncirus) cv ‘Rubidoux’ (C-20); *M. paniculata* L. Jack (orange jasmine) (C-21); *F. margarita* Lour. (kumquat) (C-22). Budwood of these genotypes was grafted onto ‘Rangpur’ lime (*C. limonia*), and plants were grown in 3-L plastic bags within a screen house.

### 3.2. Diaphorina Citri Rearing

To supply insects for bioassays, *D. citri* was reared on orange jasmine (*M. paniculata*) in a greenhouse. The reared insects were kept in 50 cm × 90 cm × 50 cm aluminum cages covered with an anti-aphid screen. Orange jasmine plants were cultivated in 0.5-L plastic bags and pruned after blooming. Fifty centimeters long stems were used for oviposition and feeding. Plants were kept in the oviposition cages for 2 to 3 weeks, after which they were replaced by new ones. Plants with eggs and nymphs were transferred to new cages (emergence cages for the adults), and emerged adults were collected and transferred to oviposition cages every 2 days. After plant removal, the cages were washed to reduce the incidence of entomopathogenic fungi. After emergence of all adults, the orange jasmine plants were removed from the cages, pruned, and sprayed with imidacloprid (Provado 200 SC^®^, Bayer S.A., São Paulo, SP, Brazil) for their future reuse in the oviposition cages.

### 3.3. Isolation and Analysis of Essential Oils

The leaves from new shoots (18.0 g in 400 mL water) of each species were submitted to steam distillation for 3 h, using a Clevenger apparatus. The essential oils obtained were extracted with CH_2_Cl_2_, dried over anhydrous Na_2_SO_4_, concentrated under vacuum and kept in the freezer.

The analysis of the oils were carried out on a GC-17A gas chromatograph (Shimadzu, Kyoto, Japan) fitted with a fused silica DB-5 (30 m × 0.25 mm ID, 0.25 μm film thickness) capillary column (Agilent J & W, Santa Clara, CA, USA) with helium as the carrier gas at a flow rate of 1.2 mL∙min^−1^. The temperature was programmed initially at 50 °C for 3 min, and then increased with a rate of 5 °C∙min^−1^ to 150 °C and increased again with a rate of 10 °C∙min^−1^ to 260 °C∙min^−1^, isotherm of 260 °C for 15 min. The injection was split and its temperature was 250 °C. The interface temperature was 280 °C. Injection volume was 1.0 μL solution in CH_2_Cl_2_. The chromatograph was coupled to a Shimadzu QP5000 mass selective detector at 70 eV. Scanning speed was 0.5 scan∙sec^−1^ from *m*/*z* 50 to 500. Identification of the components was made by determination of their retention indices relative to those of a homologous series of *n*-alkanes (C_10_-C_24_) [18], by comparison with fragmentation patterns in mass spectra with those stored on the spectrometer database and bibliography [19]. Three batches of leaves were constituted for each genotype.

### 3.4. Multivariate and Statistical Analysis

Pirouette (v 4.0, Infometrix Inc, Bothell, WA, USA) was used to process 22 spectra from 22 genotypes of *Citrus*. In total ion chromatograms (TIC) the retention times (3.5–47 min) were used as variables (rows represented the essential oil analyzed of 22 genotypes, and columns the retention times). The concentrated essential oils were prepared in triplicate. All analytical data were autoscaled to produce variables with zero means and unit standard deviation.

### 3.5. Olfactory Bioassays with the 22 Genotypes of Citrus

Bioassays were performed in a dual choice Y-tube olfactometer operated with a pre-humidified and charcoal filtered airflow from an air pump at a flow of 0.1 L∙mim^−1^. The olfactometer consisted of a Y-shaped glass tube of 3.0 cm diameter, 15 cm in length, 10 cm of arms and a 120° Y angle. Bioassays were performed in groups of 10 individuals, not sexed and aged between five and seven days with 10 replicates for each treatment: (i) control (air), (ii) essential oils (1.0 µL) and responses were recorded 15 min after the drive system, which was based on preliminary tests. In each repetition, the olfactometer was rotated 180°, avoiding possible conditioning of insects in the environment. The choice of odor sources of 22 volatile oils from 22 genotypes of *Citrus* and moist air (blank) were tested. A line was drawn on each of the two arms at 3.0 cm distance from the arm junction. Psyllids crossing the line within 15 min spells were considered to have made a choice. All assays were performed at room temperature 25 ± 1°C and 70% ± 10% RH, and the data were analyzed by paired Chi-square test (*p* ≤ 0.05). Chi-square tests were used to analyze pair-wise differences in the number of choices between the two odor sources. Differences in the total number of responsive insects between the different odor combinations were determined using a contingency table (Microsoft Excel). Index of repellency (IR) was calculated by following formula: IR = 2 × G/G + P; where G is the percentages of insects present on treated and P on control. The repellency index is classified as: values <1 repellency; 1 neutral; >1 attractant [15]. Psyllids feed only during daylight hours, and in preliminary tests they showed a preference for the afternoon light, thus the experiments were developed with light control.

## 4. Conclusions

Many pest species are exceptionally well equipped to respond to environmental stresses because of their short generation time and great reproductive potential. The use of chemical sprays to control insect creates a potent environmental stress. There are now many examples of pests that have responded by developing resistance to one or more pesticides. In addition, few new pesticides are being developed and marketed for insects because of the high cost of pesticide discovery and the necessary years of continuous and cumbersome research. Due to these challenges, investigators need to consider all possible routes to obtain novel pesticides. In conclusion, this study offers insights to develop olfactory lures for enhancing *D. citri* detection and monitoring, as well as to identify effective repellents from plants.

## Figures and Tables

**Table 1:** Effect of essential oil from 22 genotypes of *Citrus* (C-1–C-22) on repellency of *D. citri*.

*Citrus* Genotype	Index of Repellency (IR)
*C. sinensis* (sweet orange) cv. ‘Pera’ (C-1)	0.92 ± 0.17
*C. sinensis* (sweet orange) cv. ‘Natal’ (C-2)	1.29 ± 0.17
*C. sinensis* (sweet orange) cv. ‘Valencia’ (C-3)	1.20 ± 0.16
*C. sinensis* (sweet orange) cv. ‘Washington Navel’ (‘Bahia’) (C-4)	1.25 ± 0.20
*C. sinensis* (sweet orange) cv. ‘Hamlin’ (C-5)	1.04 ± 0.18
*C. reticulata* Blanco (tangerine or mandarin) cv. ‘Cravo’ (C-6)	1.11 ± 0.15
*C. reticulata* Blanco (tangerine or mandarin) cv. ‘Ponkan’ (C-7)	1.58 ± 0.17
*C. deliciosa* Tenore (mandarin) cv. ‘Mexerica-do-rio’ (C-8)	1.23 ± 0.08
*C. limettioides* Tanaka (sweet lime) cv ‘Palestine’ (C-9)	1.22 ± 0.24
*C. latifolia* Tanaka (lime) cv. ‘Tahiti’ (C-10)	1.29 ± 0.17
*C. paradisi* Mcf. (grapefruit) cv. ‘Marsh Seedless’ (C-11)	1.39 ± 0.14
*C. limon* (L.) Burm. F. (Sicilian lemon) (C-12)	1.32 ± 0.14
*C. aurantium* L. (sour orange) (C-13)	1.16 ± 0.21
*C. grandis* Osbeck (sweet pummel) (C-14)	1.15 ± 0.19
*C. medica* L. (citron) (C-15)	1.43 ± 0.18
*C. reticulata* L. × *C. sinensis* L. (tangor) ‘Murcott’ (C-16)	0.81 ± 0.16
*C. paradisi* × *P. trifoliata* (citrumelo) cv. ‘Swingle’ (C-17)	0.76 ± 0.19
*P. trifoliata* × *C. sinensis* (citrange) cv. ‘Troyer’ (C-18)	0.95 ± 0.25
*C. sunki* hort. ex Tanaka × *P. trifoliata* L. Raf. (citrandarin) ‘English’ (C-19)	1.10 ± 0.20
*P. trifoliata* L. Raf. (poncirus) cv ‘Rubidoux’ (C-20)	1.31 ± 0.21
*M. paniculata* L. Jack (orange jasmine) (C-21)	1.75 ± 0.07
*F. margarita* Lour. (kumquat) (C-22)	1.18 ± 0.20

The repellency index is classified as: values <1 repellency; 1 neutral; >1 attractant [15].

## Supplementary Materials

The following are available online at http://www.mdpi.com/1420-3049/21/6/814/s1, Figure S1-S22: GC (TIC) chromatograms of volatile oils from 22 genotypes of *Citrus*, Table S1: Constituents identified in volatile oils from 22 genotypes of *Citrus*, Table S2: Figure 1 data.

## Abbreviations

The following abbreviations are used in this manuscript:

GC-MS: Gas Chromatography-Mass Spectrometry
PCA: Principal Component Analysis
PCs: Principal Components
