# Supplementary material for: Essential Oil Variation from Twenty Two Genotypes of Citrus in Brazil—Chemometric Approach and Repellency Against Diaphorina citri Kuwayama
Source: Molecules. 2016 Jun 22;21(6):814. doi: 10.3390/molecules21060814 (PMC6272910; doi:10.3390/molecules21060814)
Supplement: Supplementary file 1 [file molecules-21-00814-s001.pdf]

Supplementary Materials: Essential Oil Variation of Twenty Two Genotypes of *Citrus* in Brazil—Chemometric Approach and Repellency Against *Diaphorina citri* Kuwayama

Moacir dos Santos Andrade, Leandro do Prado Ribeiro, Paulo Cesar Borgoni, Maria Fátima das Graças Fernandes da Silva, Moacir Rossi Forim, João Batista Fernandes, Paulo Cezar Vieira, José Djair Vendramin and Marcos Antônio Machado

1. Fiugures

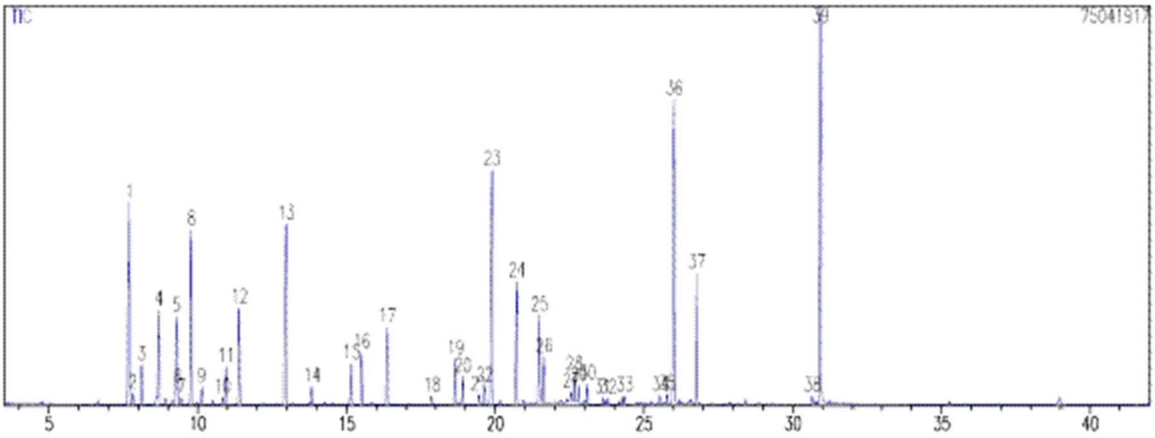

Figure S1. GC (TIC) chromatogram of volatile oils from *Citrus sinensis* cv. 'Pera' (C-1), indicating the major compounds.

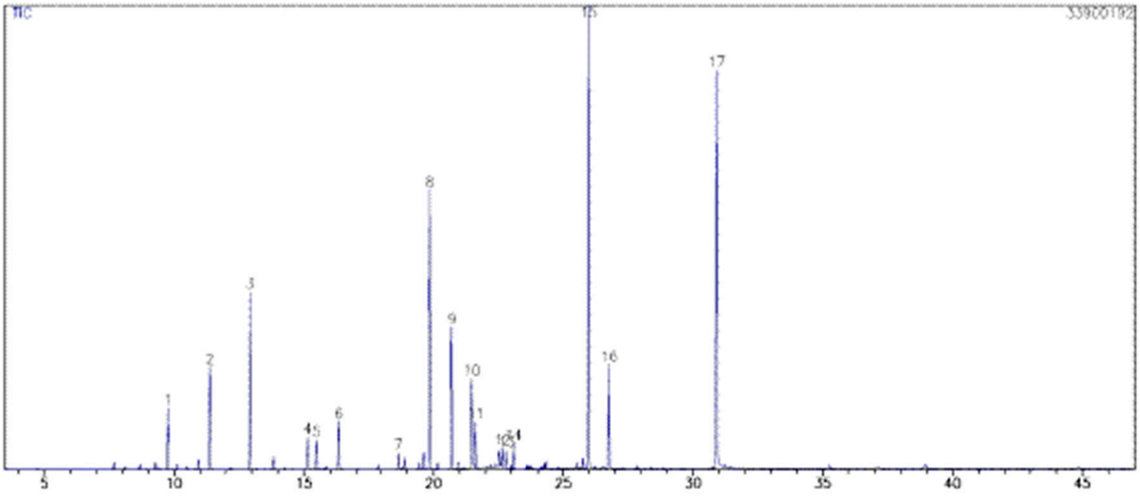

Figure S2. GC (TIC) chromatogram of volatile oils from *Citrus sinensis* cv. 'Natal' (C-2), indicating the major compounds.

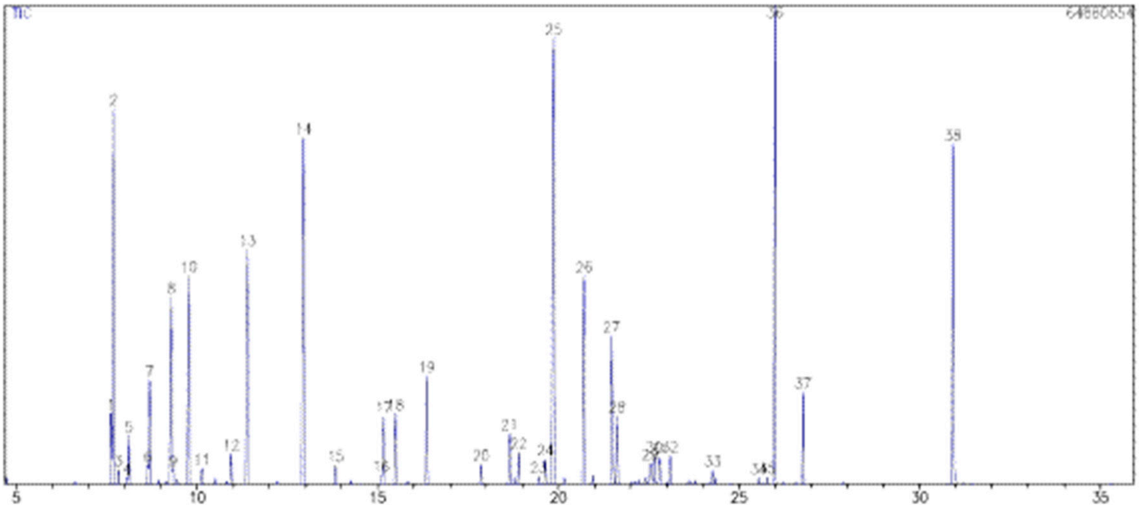

Figure S3. GC (TIC) chromatogram of volatile oils from *Citrus sinensis* cv. 'Valencia' (C-3), indicating the major compounds.

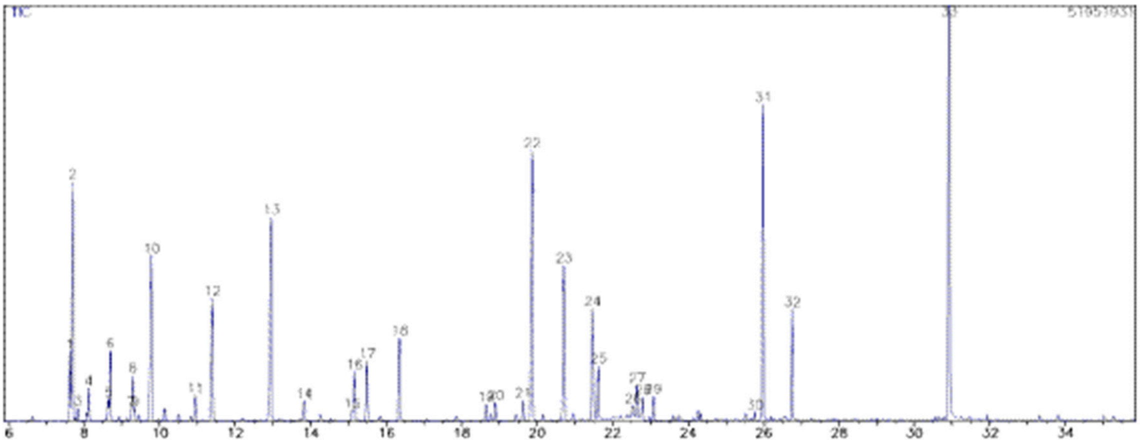

Figure S4. GC (TIC) chromatogram of volatile oils from *Citrus sinensis* cv. 'Washington Navel' ('Bahia') (C-4), indicating the major compounds.

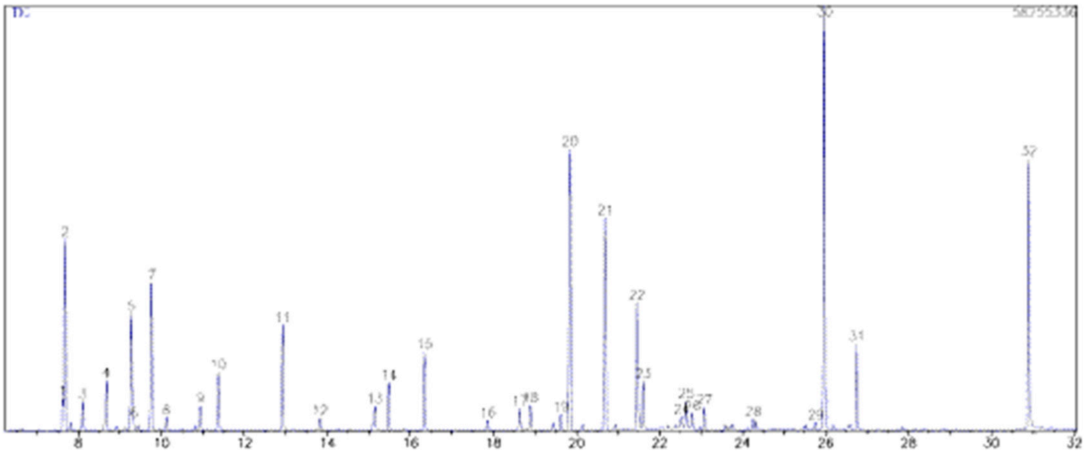

Figure S5. GC (TIC) chromatogram of volatile oils from *Citrus sinensis* cv. 'Hamlin' (C-5), indicating the major compounds.

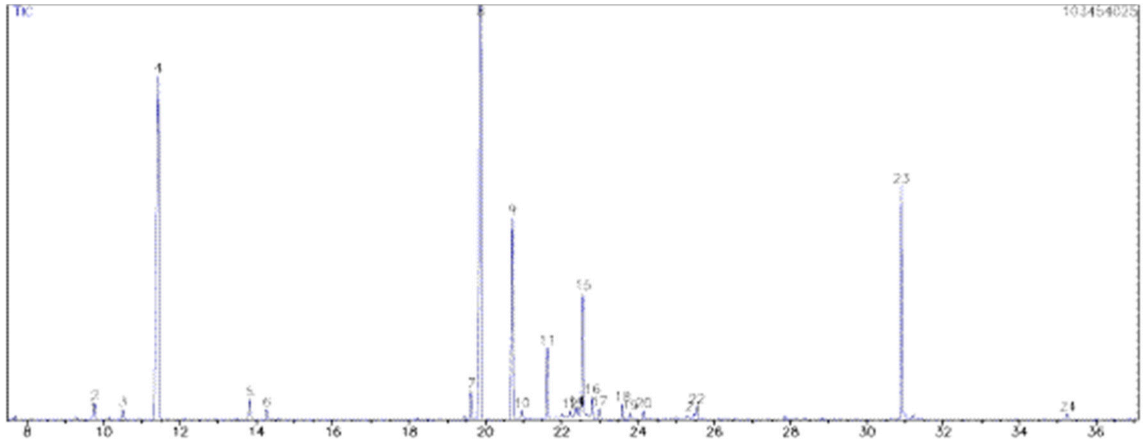

Figure S6. GC (TIC) chromatogram of volatile oils from *C. reticulata* Blanco (tangerine or mandarin) cv. 'Cravo' (C-6), indicating the major compounds.

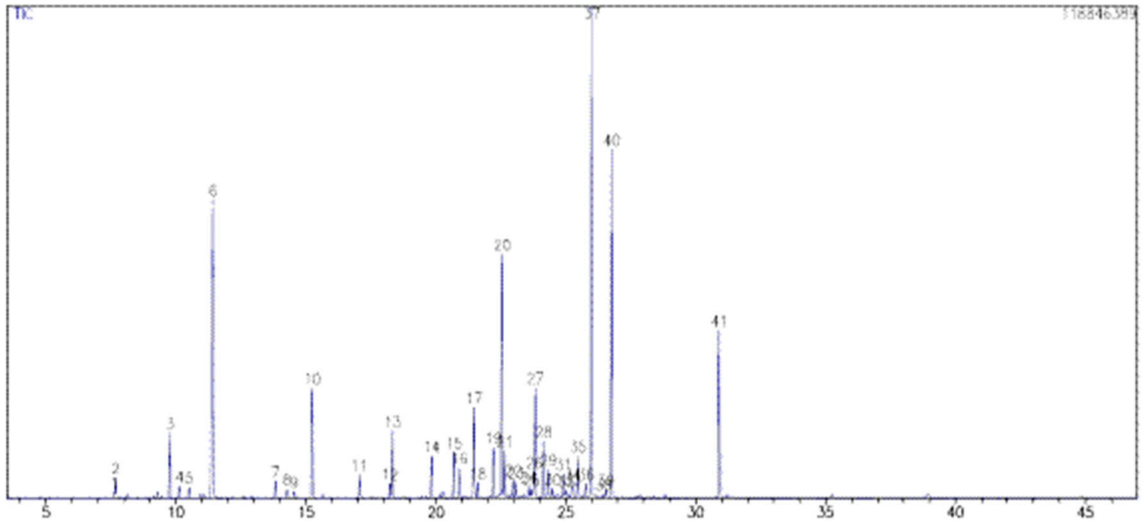

Figure S7. GC (TIC) chromatogram of volatile oils from *C. reticulata* Blanco (tangerine or mandarin) cv. 'Ponkan' (C-7), indicating the major compounds.

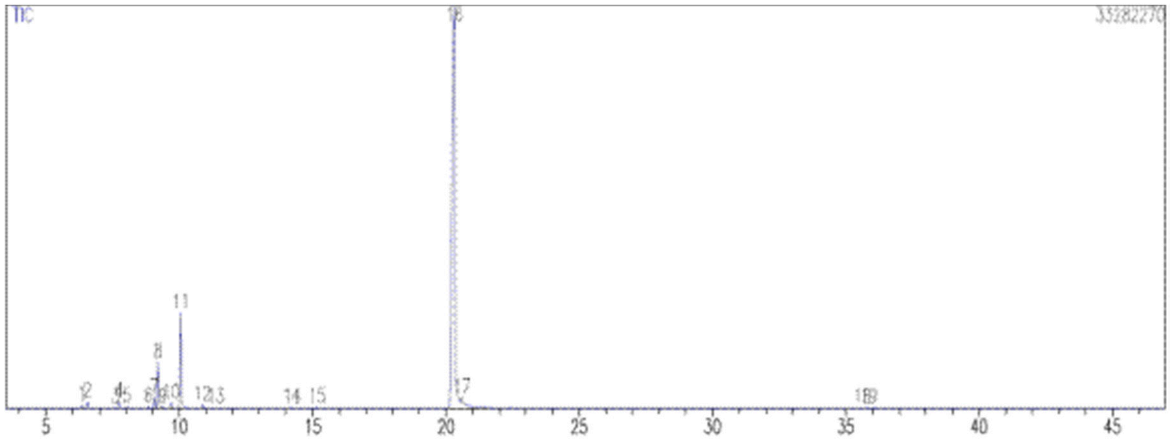

Figure S8. GC (TIC) chromatogram of volatile oils from *C. deliciosa* Tenore (mandarin) cv. 'Mexerica-do-rio' (C-8), indicating the major compounds.

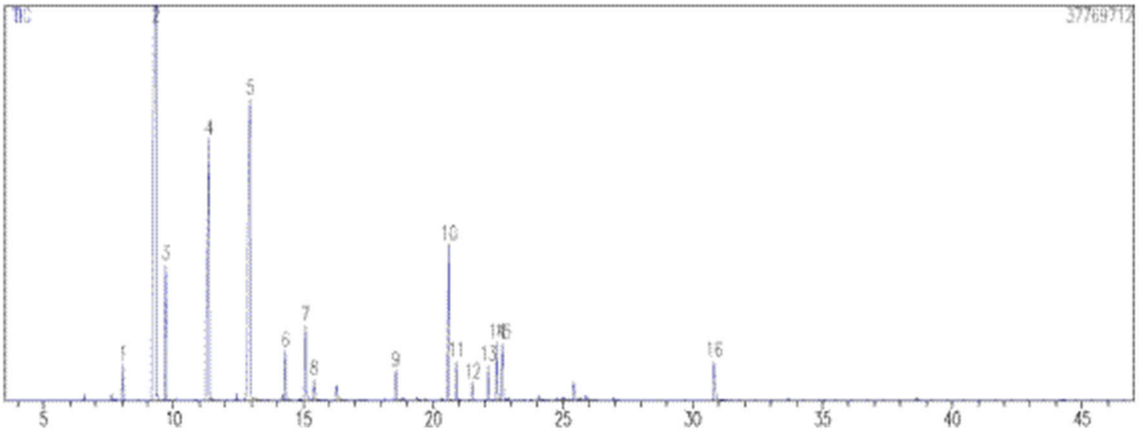

Figure S9. GC (TIC) chromatogram of volatile oils from *C. limettioides* Tanaka (sweet lime) cv 'Palestine' (C-9), indicating the major compounds.

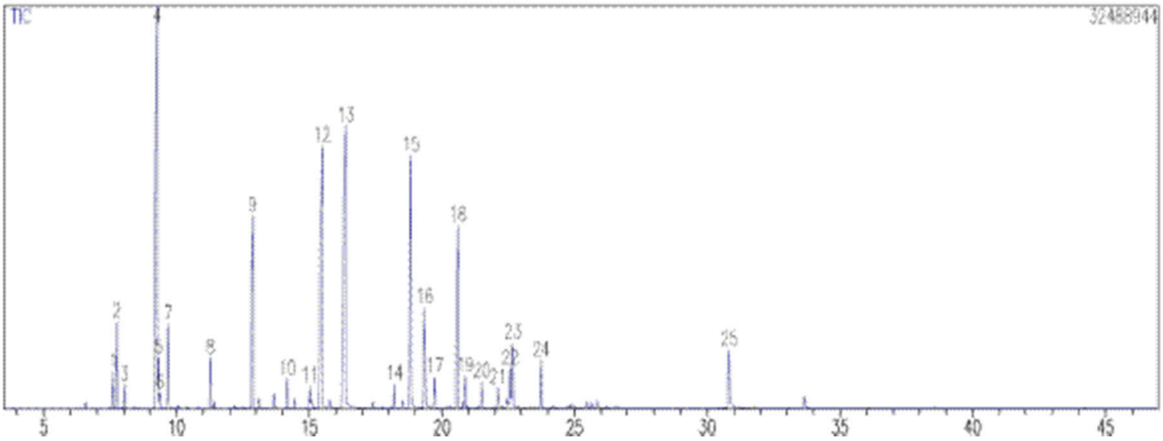

Figure S10. GC (TIC) chromatogram of volatile oils from *C. latifolia* Tanaka (lime) cv. 'Tahiti' (C-10), indicating the major compounds.

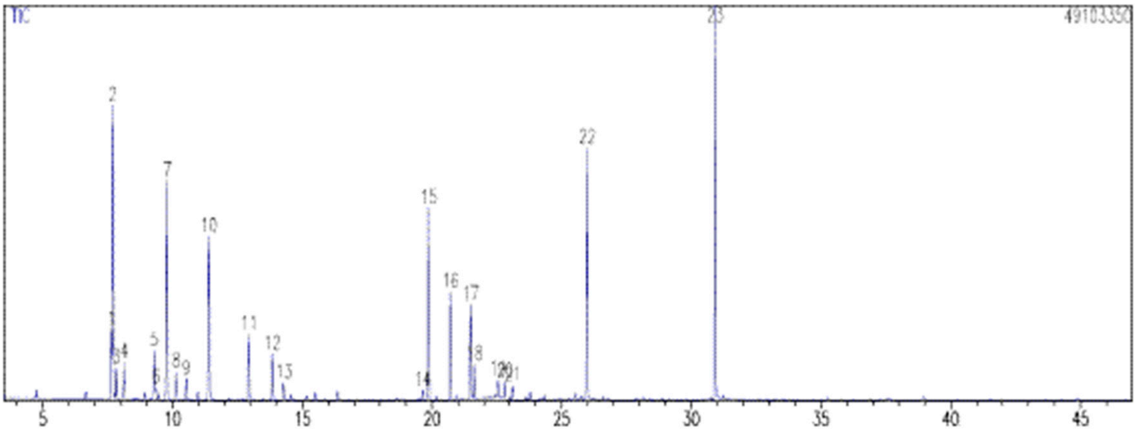

Figure S11. GC (TIC) chromatogram of volatile oils from *C. paradisi* Mcf. (grapefruit) cv. 'Marsh Seedless' (C-11), indicating the major compounds.

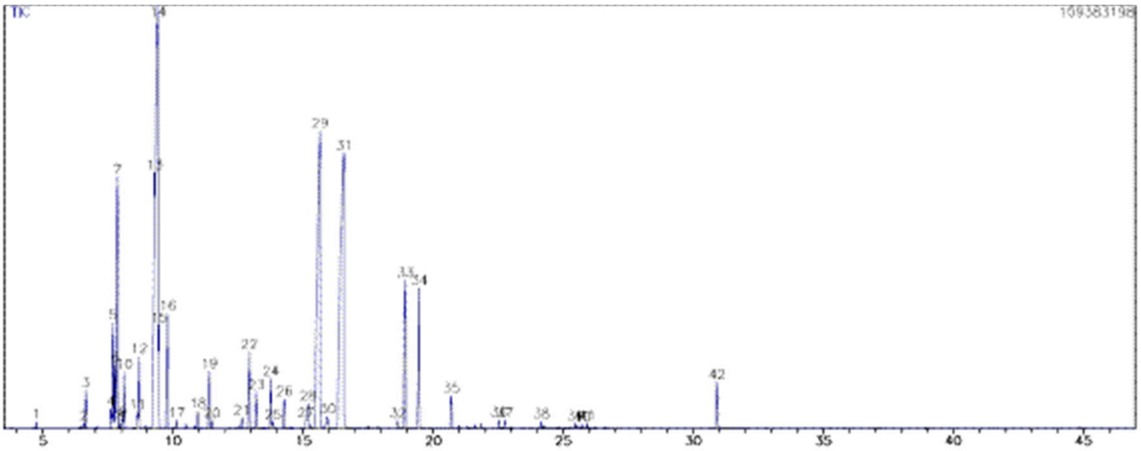

Figure S12. GC (TIC) chromatogram of volatile oils from *C. limon* (L.) Burm. F. (Sicilian lemon) (C-12), indicating the major compounds.

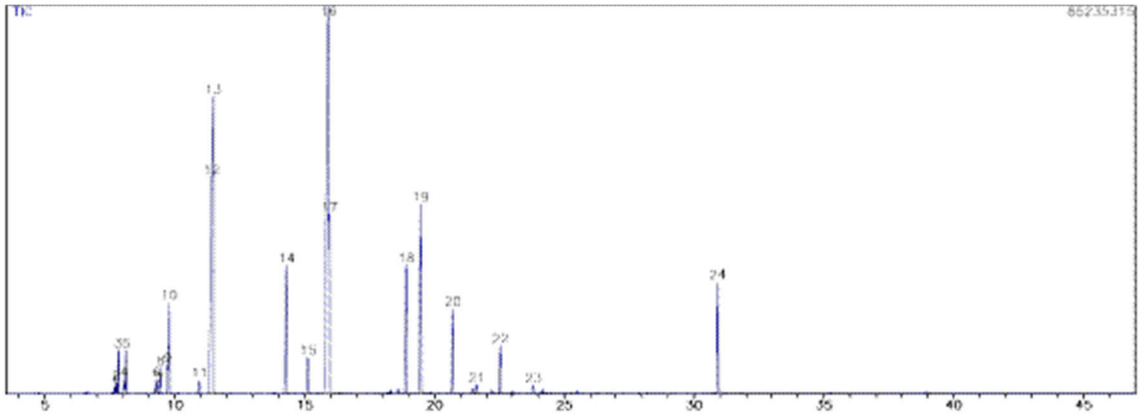

Figure S13. GC (TIC) chromatogram of volatile oils from *C. aurantium* L. (sour orange) (C-13), indicating the major compounds.

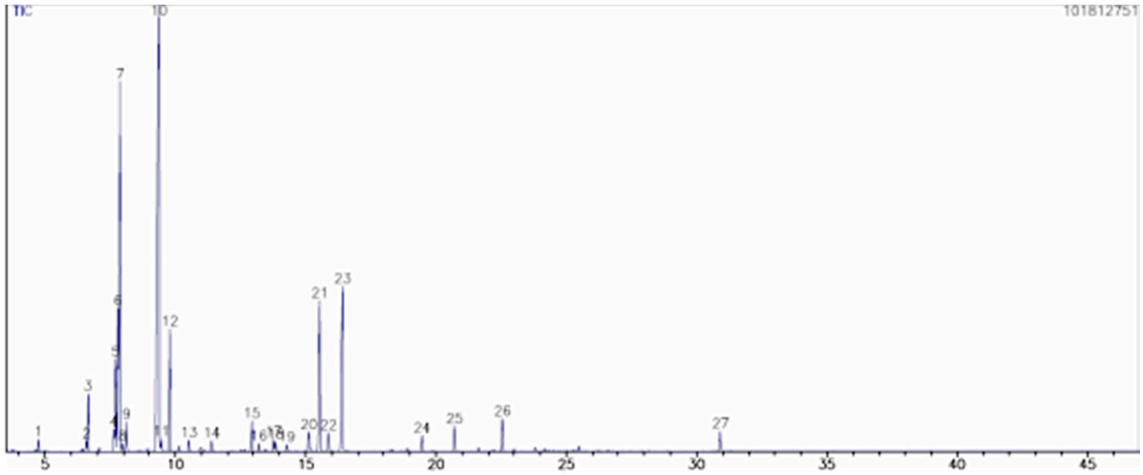

Figure S14. GC (TIC) chromatogram of volatile oils from *C. grandis* Osbeck (sweet pummel) (C-14), indicating the major compounds.

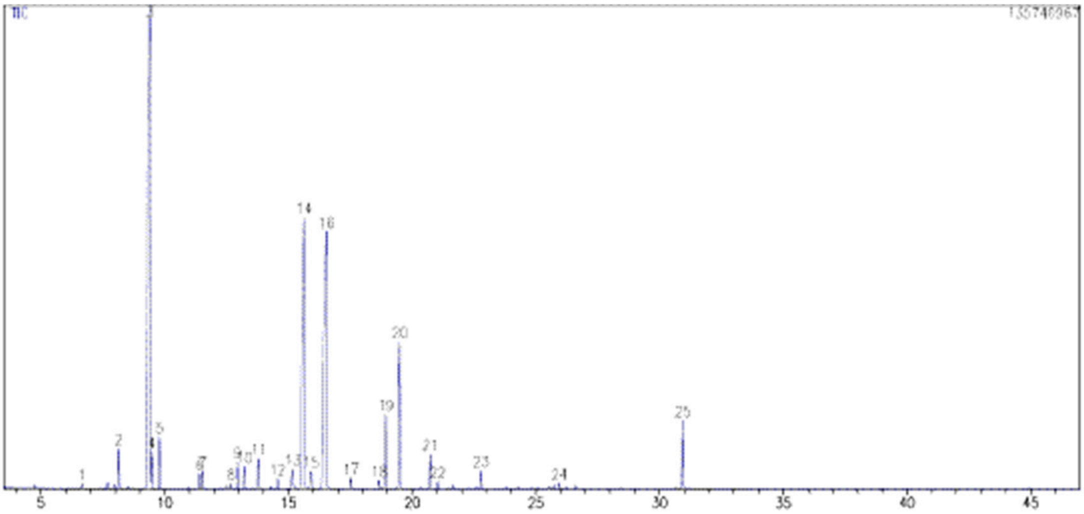

Figure S15. GC (TIC) chromatogram of volatile oils from *C. medica* L. (citron) (C-15), indicating the major compounds.

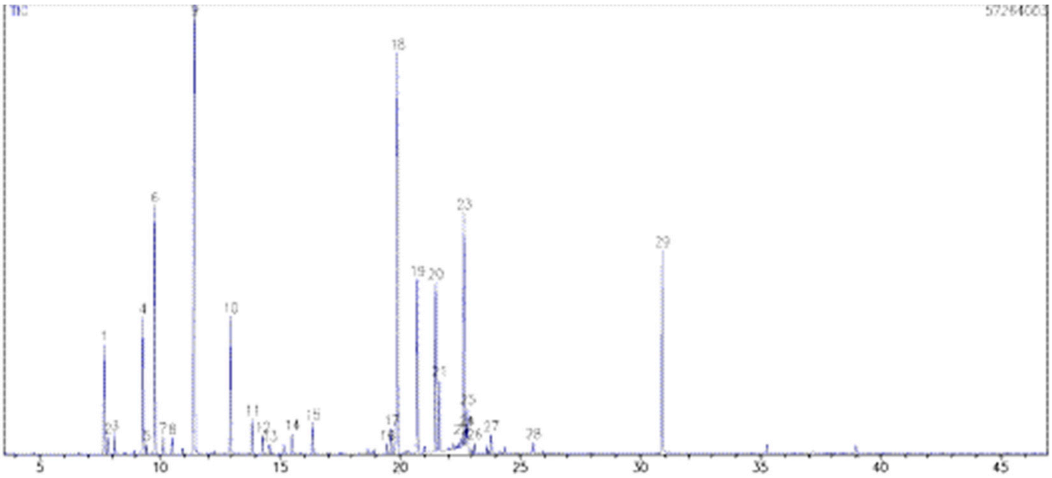

Figure S16. GC (TIC) chromatogram of volatile oils from *C. reticulata* L. x *C. sinensis* L. (tangor) 'Murcott' (C-16), indicating the major compounds.

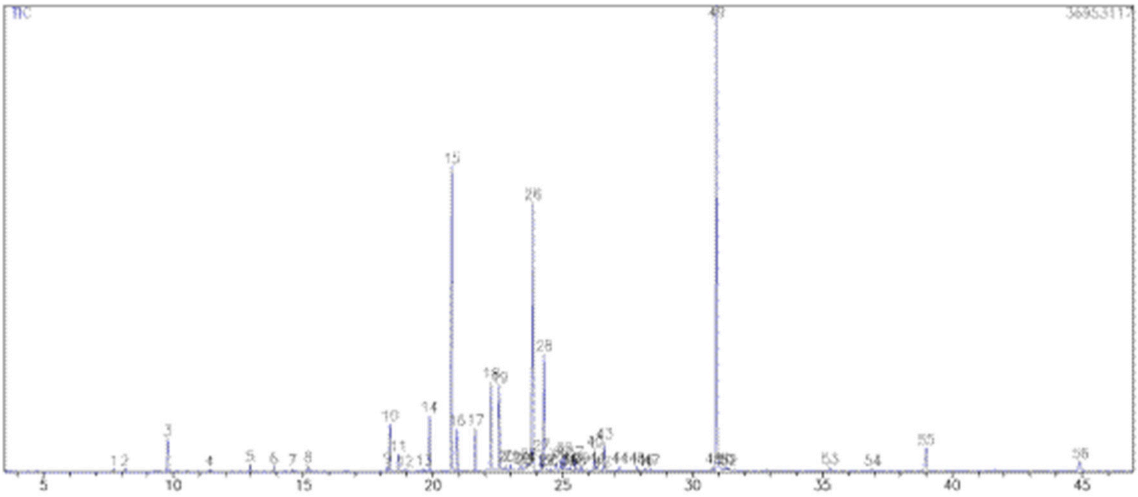

Figure S17. GC (TIC) chromatogram of volatile oils from *C. paradisi* x *P. trifoliata* (citrumelo) cv. 'Swingle' (C-17), indicating the major compounds.

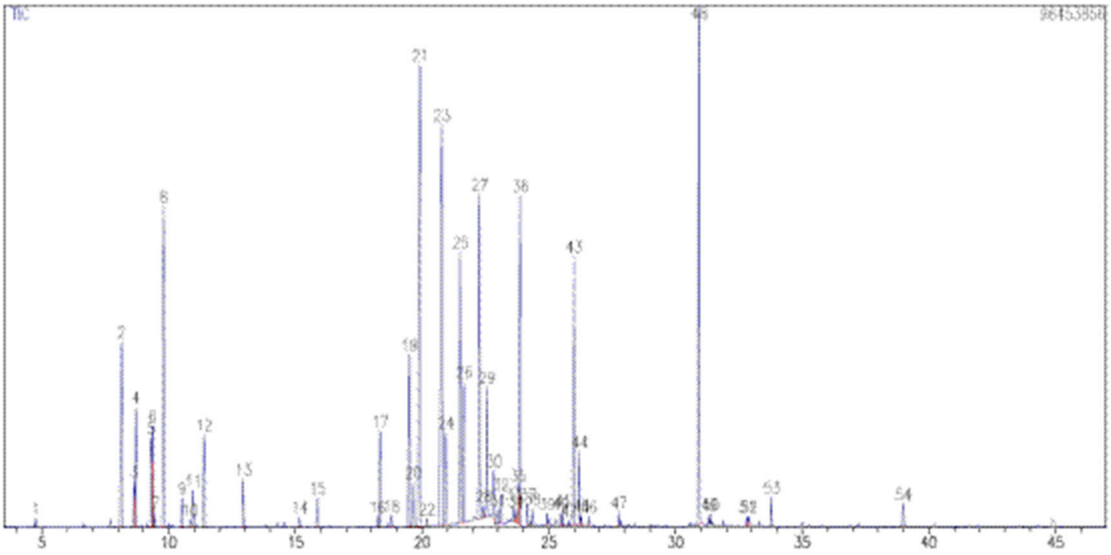

Figure S18. GC (TIC) chromatogram of volatile oils from *P. trifoliata* x *C. sinensis* (citrange) cv. 'Troyer' (C-18), indicating the major compounds.

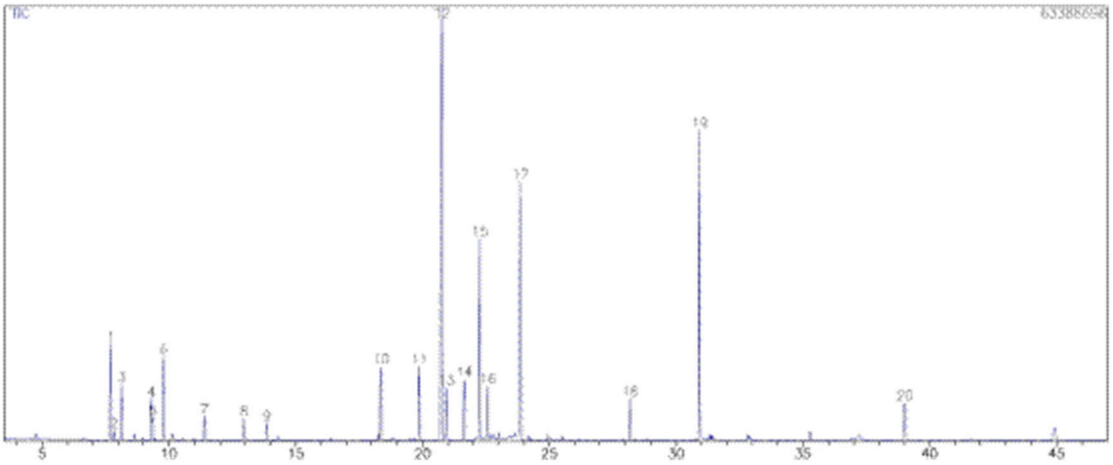

Figure S19. GC (TIC) chromatogram of volatile oils from *C. sunki* hort. ex Tanaka x *P. trifoliata* L. Raf. (citrandarin) 'English' (C-19), indicating the major compounds.

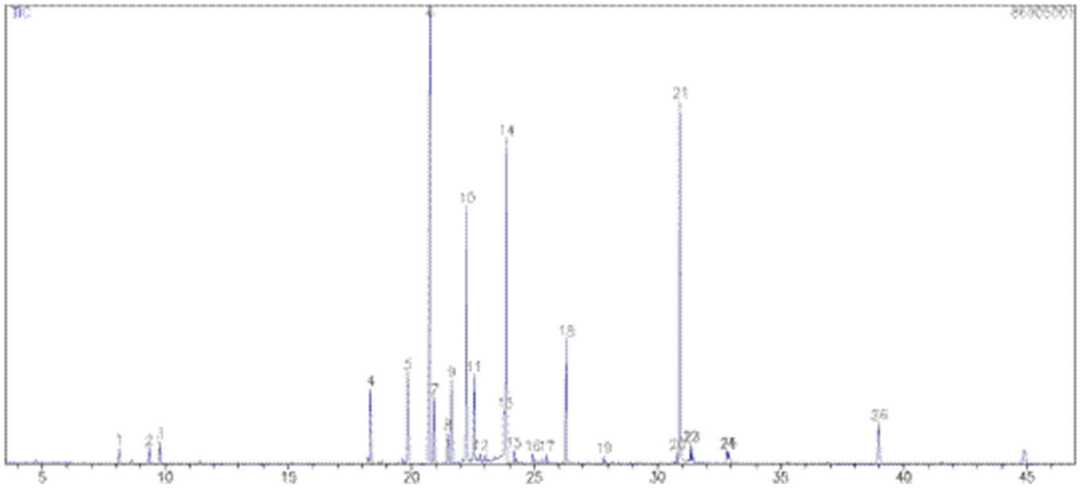

Figure S20. GC (TIC) chromatogram of volatile oils from *P. trifoliata* L. Raf. (poncirus) cv 'Rubidoux' (C-20), indicating the major compounds.

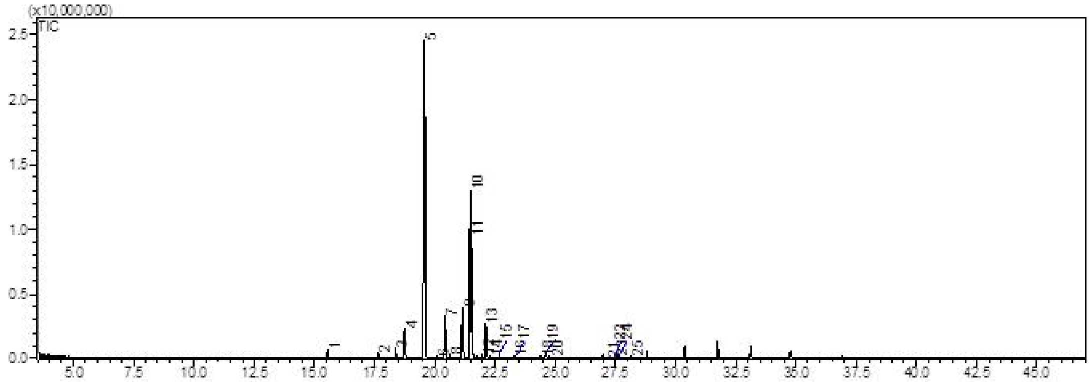

Figure S21. GC (TIC) chromatogram of volatile oils from *M. paniculata* (C-21), indicating the major compounds.

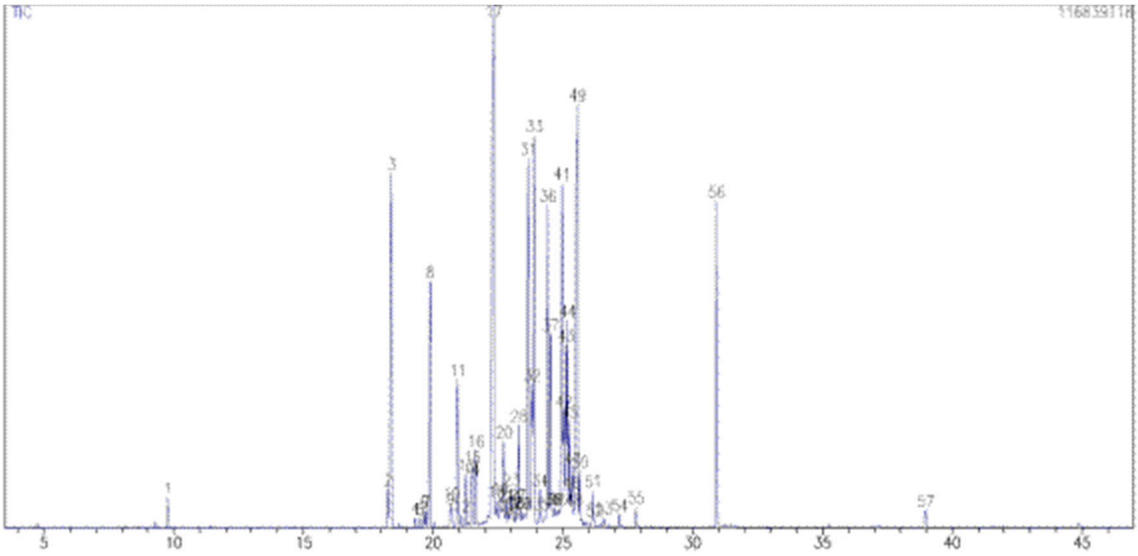

Figure S22. GC (TIC) chromatogram of volatile oils from *F. margarita* Lour. (kumquat) (C-22), indicating the major compounds.

Table S1. Essential oil composition (%) from 22 genotypes of *Citrus* (C-1–C-22).

RI	RI*	Compounds	Genotypes of *Citrus*																					
			C22 %	C18 %	C07 %	C06 %	C05 %	C03 %	C04 %	C01 %	C20 %	C19 %	C15 %	C21 %	C10 %	C09 %	C08 %	C17 %	C11 %	C02 %	C16 %	C13 %	C14 %	C12 %
**918**	**-**	NI	-	-	-	-	-	-	-	-	-	-	-	-	-	-	0.22	-	-	-	-	-	-	-
**928**	**-**	NI	-	-	-	-	-	-	-	-	-	-	-	-	-	-	-	-	-	-	-	-	0.35	-
**930**	**932**	α-Pinene (C10)	-	-	-	-	-	-	-	-	-	-	-	-	-	-	0.50	-	-	-	-	-	1.65	0.56
**966**	**975**	Sabinene (C10)	-	-	0.19	-	1.34	1.85	2.24	-	-	-	-	-	0.93	-	-	-	12.48	-	3.80	-	3.66	2.02
**966**	**-**	NI	-	-	-	-	-	-	-	-	-	-	-	-	-	-	-	-	3.23	-	-	0.34	0.77	0.33
**969**	**979**	β-Pinene (C10)	-	-	0.64	0.19	6.45	7.93	7.51	8.61	-	5.12	-	-	2.39	-	0.47	-	1.43	-	-	1.26	17.9	5.41
**972**	**-**	NI	-	-	-	-	-	0.40	0.44	-	-	0.43	-	-	-	-	-	-	-	-	0.58	-	5.68	1.05
**982**	**-**	NI	-	-	-	-	-	0.19	-	-	-	-	-	-	-	-	-	-	-	-	-	0.42	-	-
**984**	**991**	β-Myrcene (C10)	-	3.88	-	-	0.83	1.02	0.80	1.38	0.58	2.68	1.26	-	0.52	1.15	-	0.21	1.15	-	0.56	1.22	1.14	0.98
**1003**	**1002**	Carene (C10)	-	-	-	-	-	0.58	0.71	-	-	-	-	-	-	-	-	-	-	-	-	-	-	0.33
**1004**	**1003**	α-Phellandrene (C10)	-	0.88	-	-	1.54	2.18	2.15	3.14	-	-	-	-	-	-	-	-	-	-	-	-	-	1.21
**1005**	**1004**	*para*-Mentha-1(7),8-diene (C10)	-	2.37	-	-	-	-	-	-	-	-	-	-	-	-	-	-	-	-	-	-	-	-
**1018**	**-**	NI		-	-	-	-	-	-	-	-	-	-		-	-	0.79	-	-	-	-	-	-	-
**1026**	**-**	NI	-	-	-	-	-	-	-	-	-	-	-	-	-	-	-	-	-	-	-	-	-	7.20
**1026**	**1029**	Limonene (C10)	-	1.99	-	-	5.04	5.04	1.46	3.38	-	2.16	38.64	-	21.4	37.6	3.39	-	2.46	-	4.80	0.34	38.02	19.83
**1027**	**-**	NI	-		-	-	0.23	0.27	0.26	-	-	-	-	-	1.06	-	-	-	-	-	0.51	-	-	1.58
**1028**	**1030**	β-Phellandrene (C10)	-	1.59	-	-	-	-	-	-	0.51	0.94	1.00	-	0.36	-	-	-	-	-	-	0.74	0.41	-
**1043**	**1037**	(Z)-β-Ocimene (C10)	0.44	0.24	2.59	0.73	6.29	5.51	6.79	6.89	0.88	4.11	1.43	-	2.34	4.05	0.52	1.93	11.27	3.12	8.44	0.85	-	2.38
**1044**	**1050**	(E)-β-Ocimene (C10)	-	7.50	-	-	-	-	-	-	-	-	-	-	-	-	-	-	-	-	0.47	2.82	4.78	-
**1055**	**1060**	γ-Terpinene (C10)	-	-	0.45	-	0.49	0.38	-	0.57	-	-	-	-	-	-	8.07	-	1.29	-	-	-	-	-
**1068**	**1070**	Sabinene hydrate (C10)	-	-	0.42	0.47	-	-	-	-	-	-	-	-	-	-	-	-	0.92	-	0.50	-	0.39	-
**1069**	**1073**	Linalool oxide (C10)	-	0.53	-	-	-	-	-	-	-	-	-	-	-	-	-	-	-	-	-	-	-	-
**1083**	**1088**	ρ-Mentha-2.4 (8)-diene (C10)	-	0.88	-	-	1.01	0.78	0.86	1.29	-	-	-	-	-	-	0.30	-	-	-	-	-	-	0.33
**1098**	**-**	NI	-	-	-	-	-	-	-	-	-		-	-	-	-	-	-	-	-	-	-	0.36	-
**1099**	**1097**	Linalool (C10)	-	1.70	16.46	31.05	2.31	6.89	5.10	3.75	-	1.08	0.44	-	1.22	15.83	-	-	8.85	4.99	24.09	12.62	-	1.24
**1101**	**1099**	Tetrahydrolinalool (C10)	-	-	-	-	-	-	-	-	-	-	-	-	-	-	-	-	-	-	-	12.77	-	-
**1103**	**1101**	Nonanal	-	-	-	-	-	-	-	-	-	-	0.50	-	-	-	-	-	-	-	-	-	-	-
**1147**	**1153**	Citronellal (C10)	-	0.84	-	-	4.36	10.13	9.26	7.89	-	1.00	0.76	-	6.3	22.77	-	0.39	3.22	9.20	4.48	-	1.07	1.58
**1155**	**-**	NI	-	-	-	-	-	-	-	-	-	-	0.62	-	-	-	-	-	-	-	-	-	-	0.71
**1172**	**-**	NI	-	-	-	-	-	-	-	-	-	-	0.84	-	-	-	-	-	-	-	-	-	0.42	1.00
**1174**	**-**	NI	-	-	0.62	1.08	0.48	0.50	0.66	0.56	-	-	-	-	-	-	-	0.29	-	-	-	-	0.36	-
**1175**	**1177**	Terpinen-4-ol (C10)	-	-	-	-	-	-	-	-	-	0.74	-	-	-	-	-	-	2.27	-	1.13	-	-	-
**1188**	**1189**	α-Terpineol (C10)	-	-	0.32	0.47	-	-	-	-	-	-	-	-	0.80	-	-	-	0.77	-	0.60	6.14	-	0.68
**1189**	**-**	NI	-	-	-	-	-	-	-	-	-	-	-	-	-	1.52	-	-	-	-	-	-	-	-
**1196**	**-**	NI	-	-	0.21	-	-	-	-	-	-	-	-	-	-	-	-	-	-	-	-	-	-	-
**1213**	**-**	NI	-	-	-	-	-	-	-	-	-	-	-	-	0.50	-	-	-	-	-	-	-	-	-
**1216**	**-**	NI		-	-	-	-	0.24	0.28	-	-	-	-	-	-	-	-	-	-	-	-	1.61	0.68	-
**1218**	**-**	NI	-	-	-	-	0.76	1.68	1.68	1.23	-	-	0.86	-	-	-	-	0.35	-	1.43	-	-	-	-
**1219**	**1226**	Citronellol (C10)	-	-	-	-	-	-	-	-	-	-	-	-	-	2.44	-	-	-	-	-	-	-	0.64
**1221**	**1235**	Thymol methyl ether (C10)	-	-	4.11	-	-	-	-	-	-	-	-	-	-	-	-	-	-	-	-	-	-	-
**1228**	**1230**	Nerol (C10)	-	-	-	-	-	-	-	-	-	-	-	-	-	0.54	-	-	-	-	-	-	7.61	-
**1231**	**1238**	Neral (C10)	-	-	-	-	1.98	1.82	2.10	1.84	-	-	18.34	-	14.45	-	-	-	-	1.39	0.64	-	-	18.51
**1246**	**1257**	Linalyl acetate (C10)	-	-	-	-	-	-	-	-	-	-	-	-	-	-	-	-	-	-	-	33.29	-	-
**1247**	**1253**	Geraniol (C10)	-	0.47	-	-	-	-	-	-	-	-	0.76	-	-	-	-	-	-	-	-	3.33	0.80	-
**1263**	**1267**	Geranial (C10)	-	-	-	-	3.35	2.82	3.11	2.96	-	-	22.35	-	18.43	-	-	-	-	2.45	1.02	-	9.49	22.03
**1277**	**1276**	Pregeijerene B (C12)	-	-	-	-	-	-	-	-	-	-	-	0.64	-	-	-	-	-	-	-	-	-	-

**1288**	**1290**	Thymol (C10)	-	-	0.86	-	-	-	-	-	-	-	-	-	-	-	-	-	-	-	-	-	-	-
**1316**	**-**	NI	-	-	-	-	0.36	0.53	-	-	-	-	-	-	-	-	-	0.31	-	-	-	-	-	-
**1328**	**-**	NI	0.70	0.17	0.56	-	-	-	-	-	-	-	-	-	-	-	-	-	-	-	-	-	-	-
**1331**	**1338**	δ-Elemene (C15)	7.84	1.77	2.55	-	-	-	-	-	3.18	3.80	-	-	-	-	-	2.60	-	-	-	-	-	-
**1339**	**-**	NI	-	-	-	-	-	-	-	-	-	-	-	0.13	-	1.12	-	-	-	-	-	-	-	-
**1342**	**-**	NI	-	-	-	-	0.78	1.32	-	1.55	-	-	-	-	-	-	-	-	-	0.72	-	-	-	-
**1343**	**1353**	Citronellyl acetate (C10)	-	-	-	-	-	-	-	-	-	-	-	-	-	-	-	1.04	-	-	-	-	-	-
**1348**	**-**	NI	-	0.18	-	-	-	-	-	-	-	-	-	-	9.81	-	-	-	-	-	-	4.96	-	-
**1351**	**1362**	Neryl acetate (C10)	-	3.39	-	-	1.01	0.83	0.58	0.92	-	-	2.27	-	3.62	-	-	-	-	-	-	8.28	0.67	6.84
**1353**	**1348**	α-Cubebene (C15)	-	-	-	-	-	-	-	-	-	-	-	0.41	-	-	-	-	-	-	-	-	-	-
**1368**	**-**	NI	-	-	-	-	-	0.25	-	-	-	-	-	-	-	-	-	-	-	-	-	-	-	-
**1375**	**1381**	Geranyl acetate (C10)	-	-	-	1.38	0.64	0.70	0.68	0.67	-	-	5.38	-	0.81	-	-	-	-	-	0.86	-	-	-
**1379**	**1375**	α-Ylangene (C15)	0.23	0.84	-	-	-	-	-	-	-	-	-	0.97	-	-	-	-	-	-	-	-	-	-
**1383**	**1391**	β-Elemene (C15)	4.36	13.38	1.63	26.01	13.01	13.18	11.96	10.62	4.05	3.75	-	-	-	-	84.83	3.27	10.12	16.38	16.65	-	-	-
**1394**	**1388**	β-Cubebene (C15)	-	-	-	-	-	-	-	-	-	-	-	3.20	-	-	-	-	-	-	-	-	-	-
**1409**	**1409**	*Z*-Caryophyllene (C15)	-	-	-	-	-	-	-	-	-	-	-	-	6.25	5.6	0.28	-	-	-	-	-	-	-
**1412**	**-**	NI	0.30	-	-	-	-	-	-	-	-	-	-	0.09	-	-	-	-	-	-	-	-	-	-
**1414**	**1419**	(*E*)-Caryophyllene (C15)	0.32	11.24	1.79	11.01	9.65	5.80	6.24	4.89	30.41	28.85	1.16	38.47	0.87	-	-	18.71	5.67	7.96	6.43	3.18	-	0.67
**1422**	**1417**	Sesquithujene (C15)	-	-	-	-	-	-	-	-	-	-	-	-	-	1.14	-	-	-	-	-	-	-	-
**1423**	**1437**	γ-Elemene (C15)	2.46	1.97	1.15	-	-	-	-	-	2.84	2.64	-	-	-	-	-	2.55	-	-	-	-	-	-
**1424**	**1432**	β-Copaene (C15)		-	-	0.36	-	-	-	-	-	-	-	-	-	-	-	-	-	-	-	-	-	-
**1438**	**-**	NI	0.69	-	-	-	-	-	-	-	-	-	-	-	-	-	-	-	-	-	-	-	-	-
**1446**	**1441**	Aromadendrene (C15)	0.71	-	-	-	-	-	-	-	-	-	-	-	-	-	-	-	-	-	-	-	-	-
**1448**	**1443**	(*Z*)-β-Farnesene (C15)	0.80	5.56	3.79	-	5.82	4.23	4.45	3.38	1.20	-	-	-	0.66	-	-	-	5.16	5.42	5.65	-	-	-
**1454**	**1455**	α-Humulene (C15)	1.10	2.62	0.57	3.41	2.01	1.71	1.87	1.60	3.42	3.04	-	5.23		0.46	-	2.33	1.65	2.31	2.39	-	-	-
**1468**	**1466**	9-epi-(*E*)-Caryophyllene (C15)	-	-	-	-	-	-	-	-	-	-	-	0.38		1.06	-	-	-	-	-	-	-	-
**1475**	**-**	γ-Gurjunene (C15)	-	-	-	-	-	-	-	-	-	-	-	-	0.44	-	-	-	-	-	-	-	-	-
**1479**	**1480**	γ-Muurolene (C15)	-	-	1.76	0.24	-	-	-	-	-	-	-	-	-	-	-	-	-	-	-	-	-	-
**1480**	**1485**	Germacrene D (C15)	16.9	6.64	-	-	-	-	-	-	11.26	9.44	-	6.40	-	-	-	4.50	-	-	-	-	-	-
**1487**	**1490**	β-Selinene (C15)	0.26	-	-	0.39	-	-	-	-	-	-	-	-	-	-	-	-	-	-	-	-	-	-
**1489**	**-**	NI	-	0.20	-	-	-	-	-	-	-	-	-	-	-	1.49	-	-	-	-	-	-	-	-
**1493**	**1498**	α-Selinene (C15)	-	-	-	7.5	-	-	-	-	-	-	-	-	-	-	-	-	-	-	-	-	-	-
**1493**	**-**	NI	-	-	-	-	0.58	0.58	0.49	-	-	-	-	-	-	-	-	-	0.93	-	0.46	-	-	-
**1495**	**1500**	Biclyclogermacrene (C15)	-	2.37	9.41	-	-	-	-	-	3.45	2.46	-	14.55	-	-	-	4.54	-	-	-	1.56	-	-
**1498**	**-**	NI	0.51	-	-	-	1.04	0.54	0.96	0.87	-	-	-	-	1.24	-	-	-	-	0.84	-		-	-
**1499**	**1493**	α-Zingiberene (C15)	-	-	-	-	-	-	-	-	-	-	-	21.47	-	-	-	-	-	-	-	-	-	-
**1498**	**1506**	(*E.E*)-α-Farnesene (C15)	-	-	1.54	-	-	-	-	-	-	-	-	-	-	-	-	-	-	-	7.18	1.04	-	-
**1502**	**1503**	*trans*-β-Guaiene (C15)	1.50	-	-	-	-	-	-	-	-	-	-	-	-	-	-	-	-	-	-	-	-	-
**1504**	**1506**	β-Bisabolene (C15)	-	-	-	-	-	-	-	-	-	-	-	-	1.90	1.76	-	-	-	-	-	-	-	-
**1506**	**-**	NI	0.27	-	-	1.56	0.77	-	-	0.69	0.26	-	0.47	-	-	-	-	0.26	0.67	0.96	0.80	-	-	-
**1507**	**1509**	Germacrene A (C15)	-	0.85	-	-	-	-	-	-	-	-	-	-	-	-	-	-	-	-	1.41	-	-	-
**1516**	**1512**	δ-Amorphene (C15)	0.57	-	0.69	0.45	-	-	-	-	-	-	-		-	-	-	0.33	-	-	-	-	-	-
**1520**	**-**	NI	-	-	-	-	-	-	-	-	-	-	-	0.25	-	-	-	-	-	-	-	-	-	-
**1521**	**1523**	δ-cadinene (C15)	0.18	-	-	-	0.76	0.63	0.70	0.58	-	-	-	0.44	-	-	-	-	-	1.06	-	-	-	-
**1521**	**1523**	β-sesquiphellandrene (C15)	-	0.44	0.49	-	-	-	-	-	-	-	-	4.09	-	-	-	-	0.53	-	-	-	-	-
**1532**	**-**	NI	1.52	-	-	-	-	-	-	-	-	-	-		-	-	-	-	-	-	-	-	-	-
**1542**	**1534**	*trans*-Cadina-1.4-diene (C15)	-	-	-	-	-	-	-	-	-	-	-	0.27	-	-	-	-	-	-	-	-	-	-
**1543**	**-**	NI	0.29	-	-	-	-	-	-	-	-	-	-	-	-	-	-	0.25	-	-	-	-	-	-
**1548**	**1550**	Elemol (C15)	6.83	-	0.30	0.61	-	-	-	-	-	-	-	0.84	-	-	-		-	-	-	-	-	-
**1549**	**-**	NI	-	0.29	-	-	-	-	-	-	-	-	-	--	-	-	-	0.26	-	-	-	-	-	-
**1552**	**-**	NI	-	0.25	0.27	-	-	-	-	-	-	-	-	-	-	-	-	0.68	-	-	-	-	-	-

**1557**	**-**	NI	-	-	0.95	-	-	-	-	-	-	-	-	-	1.55	-	-	-	-	-	-	-	-	-
**1559**	**1563**	(*E*)-Nerolidol (C15)	1.91	0.73	-	-	-	-	-	-	1.73	-	-	-	-	-	-	-	-	-	0.58	-	-	-
**1560**	**1561**	Germacrene B (C15)	7.63	6.64	3.58	-	-	-	-	-	13.93	12.02	-	-	-	-	-	16.76	-	-	-	-	1.15	-
**1577**	**1576**	Germacrene D-4-ol (C15)	0.43	0.32	1.78	0.32	-	-	-	-	0.39	-	-	-	-	-	-	0.81	-	-	-	-	-	-
**1583**	**-**	NI	-	-	-	-	0.37	0.35	-	-	-	-	-	-	-	-	-	-	-	-	-	-	-	-
**1583**	**1578**	Spathulenol (15)	-	-	-	-	-	-	-	-	-	-	-	0.28	-	-	-	-	-	-	-	-	-	-
**1584**	**1583**	Caryophyllene oxide (C15)	-	-	-	-	-	-	-	-	-	-	-	-	-	-	-	6.07	-	-		-	-	-
**1587**	**1579**	*trans*-Sesquisabinene hydrate	-	0.31	0.92	-	-	-	-	-	-	-	-	-	-	-	-	-	-	-	-	-	-	-
**1595**	**-**	NI	5.01	-	0.30	-	-	-	-	-	-	-	-	0.42	-	-	-	-	-	-	-	-	-	-
**1597**	**1601**	Guaiol (C15)	3.51	-	-	-	-	-	-	-	-	-	-	-	-	-	-	-	-	-	-	-	-	-
**1612**	**-**	NI	-	-	-	-	-	-	-	-	-	-	-	0.15	-	-	-	0.50	-	-	-	-	-	-
**1621**	**-**	NI	-	-	0.68	-	-	-	-	-	0.27	-	-	0.12	-	-	-	-	-	-	-	-	-	-
**1625**	**-**	NI	6.30	-	-	-	-	-	-	-	-	-	-	-	-	-	-	0.49	-	-	-	-	-	-
**1628**	**-**	NI	1.82	-	0.32	-	-	-	-	-	-	-	-	-	-	-	-	-	-	-	-	-	-	-
**1630**		NI	-	-	-	-	-	-	-	-	-	-	-	-	-	-	-	0.74	-	-	-	-	-	-
**1635**	**1631**	Eremoligenol (C15)	2.76	-	-	-	-	-	-	-	-	-	-	-	-	-	-	-	-	-	-	-	-	-
**1638**	**-**	NI	2.34	-	-	-	-	-	-	-	-	-	-	0.13	-	-	-	-	-	-	-	-	-	-
**1642**	**-**	NI	-	-	0.28	-	-	-	-	-	-	-	-	-	-	-	-	0.30	-	-	-	-	-	-
**1643**	**1648**	Agarospirol (C15)	1.23	-	-	-	-	-	-	-	-	-	-	-	-	-	-	-	-	-	-	-	-	-
**1644**	**-**	NI	-	-	0.46	-	-	-	-	-	-	-	-	-	-	-	-	-	-	-	-	-	-	-
**1647**	**1646**	Cubenol (C15)	0.45		-	-	-	-	-	-	-	-	-	0.32	-	-	-	-	-	-	-	-	-	-
**1651**	**-**	NI	0.69		-	-	-	0.14	-	-	-	-	-	-	-	-	-	-	-	-	-	-	-	-
**1656**	**1654**	α-Eudesmol (C15)	-	0.21	-	0.34	-	-	-	-	-	-	-	-	-	-	-	-	-	-	-	-	-	-
**1661**	**-**	NI	-	0.24	-	0.54	-	-	-	-	-	-	-	-	-	-	-	-	-	-	-	-	-	-
**1663**	**1654**	α-Cadinol (C15)	10.51	-	1.43	-	-	-	-	-	0.26	-	-	-	-	-	-	0.58	-	-	-	-	-	-
**1668**	**-**	NI	0.60	-	-	-	-	-	-	-	-	-	-	0.39	-	-	-	-	-	-	-	-	-	-
**1675**	**-**	NI	-	-	0.43	-	0.24	0.16	0.19	-	-	-	-	-	-	-	-	0.26	-	-	-	-	-	-
**1691**	**1700**	β-Sinensal (C15)	-	4.40	19.16	11.18	14.70	10.01	10.09	11.62	-	-	-	-	-	-	-	-	10.22	20.97	-	-	-	-
**1701**	**-**	NI	0.44	-	-	-	-	-	-	-	-	-	-	-	-	-	-	-	-	-	-	-	-	-
**1705**	**1701**	(2*Z*,6*Z*)-Farnesol (C15)	-	1.05	-	-	-	-	-	-	-	-	-	-	-	-	-	0.84	-	-	-	-	-	-
**1710**	**1718**	(2*Z*,6*E*)-Farnesol (C15)	-	-	-	-	-	-	-	-	4.05	-	-	-	-	-	-	-	-	-	-	-	-	-
**1714**		NI	-	-	-	-	-	-	-	-	-	-	-	-	-	-	-	0.33	-	-	-	-	-	-
**1729**	**-**	NI	-	-	0.29	-	-	-	-	-	-	-	-	-	-	-	-	-	-	-	-	-	-	-
**1734**	**1725**	(2*E*,6*E*)-Farnesol (C15)	-	-	-	-	-	-	-	-	-	-	-	-	-	-	-	1.37	-	-	-	-	-	-
**1746**	**1757**	α-Sinensal (C15)	-	-	11.06	-	2.77	1.78	2.95	3.44	-	-	-	-	-	-	-	-	-	4.38	-	-	-	-
**1877**	**-**	NI	-	-	-	-	-	-	-	-	-	-	-	0.10	-	-	-	-	-	-	-	-	-	-
**1926**	**-**	NI	-	-	-	-	-	-	-	-	-	-	-	0.12	-	-	-	-	-	-	-	-	-	-
**2094**		NI	-	-	-	-	-	-	-	-	0.31	-	-	-	-	-	-	-	-	-	-	-	-	-
**2107**	**1943**	Phytol (C20)	3.34	8.02	4.93	-	9.03	6.48	11.98	12.1	12.10	11.26	1.55	-	1.94	1.46	-	19.54	14.97	16.41	4.87	2.92	0.57	0.75
**2148**	**-**	NI	-	-	-	-	-	-	-	-	0.46	-	-	-	-	-	-	-	-	-	-	-	-	-
**2155**	**-**	NI	-	-	-	-	-	-	-	-	0.44	-	-	-	-	-	-	-	-	-	-	-	-	-

RI* Adams 2007 [19]; NI: Not identified; C10, C15 and C20: mono-, sesqui-, and diterpenes, respectively. C-1–C22: see Material and Methods-Plant material.

Table S2. The score scatterplot obtained according to the two major groups.

Genotypes of *Citrus*	Factor 1 (45.4%)	Factor 2 (13.1%)
c01	7.604582	−11.1499
c02	−21.0289	−10.019
c03	25.2457	−3.65029
c04	6.412549	−13.5975
c05	31.77869	−4.24454
c06	58.48798	−1.97973
c07	56.91939	−8.75231
c08	−86.213	−2.40348
c09	−80.4554	−0.37896
c10	−78.7388	−1.38268
c11	−17.6849	−5.82211
c12	−17.8197	14.655
c13	−17.9494	0.02866
c14	−2.34417	7.285903
c15	16.04557	65.93625
c16	−19.6212	−5.87912
c17	−28.6574	−9.67238
c18	73.23972	−26.7951
c19	23.53946	44.26681
c20	84.56993	39.76504
c21	−57.9369	3.004699
c22	44.60608	−69.2152
